# Vascular Endothelial Growth Factor A (*VEGFA*) Gene Polymorphisms Have an Impact on Survival in a Subgroup of Indolent Patients with Chronic Lymphocytic Leukemia

**DOI:** 10.1371/journal.pone.0101063

**Published:** 2014-06-27

**Authors:** Carol Lozano-Santos, Jimena Martinez-Velasquez, Belen Fernandez-Cuevas, Natividad Polo, Belen Navarro, Isabel Millan, Jose Miguel Garcia, Rosa Collado, Pedro Sanchez-Godoy, Felix Carbonell, Jose Antonio Garcia-Vela, Jose Antonio Garcia-Marco, Natalia Gomez-Lozano

**Affiliations:** 1 Department of Hematology, Hospital Universitario Puerta de Hierro Majadahonda & Instituto de Investigación Puerta de Hierro Majadahonda (IDIPHIM), Madrid, Spain; 2 Department of Statistics, Hospital Universitario Puerta de Hierro Majadahonda & Instituto de Investigación Puerta de Hierro Majadahonda (IDIPHIM), Madrid, Spain; 3 Group of Immunogenetics, Hospital Universitario Puerta de Hierro Majadahonda & Instituto de Investigación Puerta de Hierro Majadahonda (IDIPHIM), Madrid, Spain; 4 Group of Oncology, Hospital Universitario Puerta de Hierro Majadahonda & Instituto de Investigación Puerta de Hierro Majadahonda (IDIPHIM), Madrid, Spain; 5 Department of Hematology, Consorcio Hospital General Universitario de Valencia, Valencia, Spain; 6 Department of Hematology, Hospital Severo Ochoa de Madrid, Madrid, Spain; 7 Department of Hematology, Hospital Universitario Ramón y Cajal de Madrid, Madrid, Spain; National Cancer Center, Japan

## Abstract

Vascular endothelial growth factor (VEGF)-mediated angiogenesis contributes to the pathogenesis of B-cell chronic lymphocytic leukaemia (CLL). We investigated the impact of *VEGFA* gene diversity on the clinical outcome of patients with this disease. A *VEGFA* haplotype conformed by positions rs699947 (–1540C>A), rs833061 (–460T>C) and rs2010963 (405C>G) and two additional single-nucleotide polymorphisms (SNPs), rs3025039 (936C>T) and rs25648 (1032C>T), were analysed in 239 patients at the time of their CLL diagnosis. Here, we showed that homozygosity for rs699947/rs833061/rs2010963 ACG haplotype (ACG^+/+^ genotype) correlated with a reduced survival in CLL patients (ACG^+/+^ vs other genotypes: HR = 2.3, p = 0.002; recessive model). In multivariate analysis, the ACG^+/+^ genotype was identified as a novel independent prognostic factor (HR = 2.1, p = 0.005). Moreover, ACG homozygosity subdivided patients with CLL with otherwise indolent parameters into prognostic subgroups with different outcomes. Specifically, patients carrying the ACG^+/+^ genotype with mutated *IgV_H_*, very low and low-risk cytogenetics, initial clinical stage, CD38 negative status or early age at diagnosis showed a shorter survival (ACG^+/+^ vs other genotypes: HR = 3.5, p = 0.035; HR = 3.4, p = 0.001; HR = 2.2, p = 0.035; HR = 3.4, p = 0.0001 and HR = 3.1, p = 0.009, respectively). In conclusion, *VEGFA* ACG^+/+^ genotype confers an adverse effect in overall survival in CLL patients with an indolent course of the disease. These observations support the biological and prognostic implications of *VEGFA* genetics in CLL.

## Introduction

A significant variability in the clinical course of B-cell chronic lymphocytic leukemia (CLL) exists as a result of multiple different pathogenic mechanisms. B cell receptor repertoire skewing and stereotypy and differences in the mutational status of the *IgV_H_* gene demonstrate an antigen-driven process. Multiple genetic lesions associated with CLL (del13q14, trisomy 12, *TP53* deletion, *ATM* deletion, *NOTCH1* and *SF3B1* mutations and others) contribute to the initiation and progression of this leukemia. Recently, there has been a growing interest in determining the impact of microenvironmental interactions, such as angiogenesis, in the pathogenesis and progression of CLL.

Vascular endothelial growth factor (VEGF) is a pro-angiogenic factor with multiple roles in tumour formation that is involved in the pathophysiology of many hematologic disorders, including CLL. Several reports have shown an enhanced microvessel density in the bone marrow and lymph nodes in patients with CLL, as a result of a VEGF-dependent angiogenesis associated with an advanced stage of disease [1]–[3]. Furthermore, it has also been reported that the resistance to apoptosis of leukemic cells in CLL is mediated by VEGF-dependent autocrine and paracrine mechanisms of cell survival [4]–[7]. In addition to the angiogenic and antiapoptotic effects on CLL cells, VEGF regulates CLL cell motility [8], [9] and the microenvironment-tumor interactions [10], [11].

Considerable variation in VEGF expression exists among individuals. However, elevated VEGF levels in the serum or plasma of CLL patients positively correlate with disease progression [12] and such patients are more likely to progress rapidly to a more advanced stage of disease [13]. In addition, high levels of expression of one of its receptors, VEGFR2, correlate with shortened survival [14].

*VEGFA* is a gene comprised of eight coding exons and several alternative spliced forms that maps to chromosome region 6p1.2. Genetic polymorphisms have been identified outside of the coding region in the 5′ and 3′ flanking regions, and these polymorphisms seem to have an influence on gene expression. SNPs rs699947 (–1540C>A) and rs833061 (–460T>C) reside in the promoter region, rs2010963 (405C>G) and rs25648 (1032C>T) in the 5′UTR and rs3025029 (1689C>T) in the 3′UTR. These polymorphisms have been associated with a variation in the levels of VEGF protein [15]–[18] and predisposition to cancer development and progression [19]–[23].

Given the reported association of VEGF levels with certain clinical conditions in this leukemia, the present study evaluated whether an association exists between *VEGFA* genetic variability and its predictive value in determining the prognosis of CLL.

## Materials and Methods

### Study population

Two hundred and thirty-nine consecutive patients with newly diagnosed CLL from four Hospitals belonging to Grupo GLIMCE in Spain (Hospital Puerta de Hierro Majadahonda, Hospital de Getafe, Hospital Severo Ochoa [Madrid, Spain] and Hospital General [Valencia, Spain]) were enrolled in this retrospective study. Also, 183 age and gender-matched control individuals from the Blood Bank Department of Hospital Puerta de Hierro were analysed in order to evaluate the characteristic distribution of *VEGFA* single nucleotide polymorphisms (SNPs) in Spanish population from the same area. The diagnosis of CLL was based upon standard morphologic and immunophenotypic criteria. Written informed consent was given by participants for their clinical records to be used in this study. This project was approved by the Ethics Committee of Hospital Puerta de Hierro (Comité Ético de Investigación Clínica Hospital Puerta de Hierro Majadahonda). Progression of disease was defined according to NCI-Guidelines criteria [24].

The patient characteristics are summarised in Table 1. Genetic abnormalities were detected by conventional cytogenetics and FISH analysis and stratified as follows: very low-risk (deletion 13q), low-risk (trisomy 12and normal karyotype) and intermediate-risk (deletion 11q) and high-risk (*TP53* deletion) [25]. The mutational status of the *IgV_H_* gene was analysed and classified according to ERIC recommendations [26]. CD38 and ZAP-70 expression was determined by flow cytometry.

**Table 1:** Clinical and molecular characteristics of the CLL patients.

*Variable*		*N (%)*
Gender	Male	136 (56.9)
	Female	103 (43.1)
Age	<65	116 (48.5)
	>65	123 (51.5)
Binet stage	A	177 (74.1)
	B	51 (21.3)
	C	11 (4.6)
RAI stage	0	116 (48.5)
	I	54 (22.6)
	II	56 (23.4)
	III	6 (2.5)
	IV	7 (2.9)
Treatment	Yes	106 (44.4)
	No	132 (55.2)
	n.d.	1 (0.4)
Status	Dead	81 (33.9)
	Alive	158 (66.1)
Cause of death	Disease	73 (76.8)
	Other	8 (8.4)
Richter	Yes	16 (6.7)
	No	223 (93.3)
*Molecular data*		
Genetic lesions		
	Very low-risk (del13q)	67 (28.0)
	Low-Risk (NC, +12)	116 (48.5)
	Intermediate-risk (del11q)	20 (8.4)
	High-risk (Δ*TP53*)	36 (15.1)
CD38 expression	Negative	178 (74.5)
	Positive	32 (13.4)
	n.d.	29 (12.1)
*ZAP-70*	Negative	60 (25.1)
	Positive	50 (20.9)
	n.d.	129 (54.0)
*IgV_H_ genes*	M	98 (41)
	UM	124 (51.9)
	n.d.	17 (7.1)

NC: normal karyotype, n.d: not determined.

### Genotyping of *VEGFA* polymorphisms

DNA was extracted from peripheral blood cells using either DNAzol (MRC, Cincinnati, OH) or a Maxwell 16 Blood DNA purification kit (Promega Corp. Madison, CA). *VEGFA* rs699947 (–1540C>A), rs833061 (–460T>C), rs2010963 (405C>G), rs25648 (1032C>T) and rs3025039 (1689C>T) genotyping was performed using TaqMan MGB probes (Applied Biosystems, Foster City, CA). All reactions were performed in a 10 µL PCR reaction with 20 ng of genomic DNA. Allelic discrimination was determined using 7500 Real Time PCR (Applied Biosystems). To validate the genotyping method and haplotype estimation, selected informative samples were analysed by DNA sequencing in a 3130xl Genetic Analyzer Sequencer (Applied Biosystems). Polymorphic positions were named relative to the reference sequence [Genbank: NM_001171630,1].

### Statistical Analysis

Individual haplotypes and their frequencies were estimated based upon a Bayesian algorithm using the Phase program (available at http://www.stat.washington.edu/stephens/phase.html [27]). Linkage disequilibrium (LD) analysis was performed using Haploview software. Pearson’s χ^2^ test was used to evaluate differences in the distribution of *VEGFA* SNPs alleles, haplotypes or genotypes between subgroups of patients based upon clinical or laboratory parameters.

Odds Ratio (OR) values (95% confidence interval) for the relative risks were calculated for alleles, genotypes and haplotypes. Additive, dominant (major homozygous plus heterozygous vs minor homozygous) and recessive (major homozygous vs heterozygous plus minor homozygous) models were applied to detect associations between genotypes and the clinical and molecular variables.

OS (overall survival) was defined as the interval between the date of diagnosis and the time of death due to CLL. The OS times of patients alive at last follow-up or lost to follow-up were censored. The association between the cumulative probability of OS among genotypes or haplotypes was calculated according to the Kaplan-Meier method, while significant differences between survival curves were evaluated with Mantel’s log-rank test. Ten variables were screened by univariate analysis. Because some chromosomal aberrations resemble other molecular prognostic factors included as covariates (deletion 11q and deletion 17p corresponds to Δ*ATM* and Δ*TP53*, respectively), these were not included in the multivariate Cox model. CD38 and ZAP-70 were also excluded due to their elevated rate of missing values. All other variables were included in the adjusted analysis to verify their independent prognostic value. Statistical data analyses were performed using the Statistical Package for the Social Sciences (SPSS) software (SPSS 15, Chicago, IL, USA).

## Results

### Characteristics of *VEGFA* polymorphisms in the patient cohort

We analysed the genotypes of five SNPs of the *VEGFA* gene, rs699947 (–1540C>A), rs833061 (–460T>C) rs2010963 (405C>G), rs3025039 (936C>T) and rs25648 (1032C>T), on the basis of their previous description as genetic markers in other cancers and/or their potential effect on gene expression.

The cohort was comprised of 239 CLL patients with a mean age of 68±12 years and a male:female ratio of 1.32∶1. Minor allele frequencies of *VEGFA* polymorphisms in our CLL cohort were >5% and are summarized in Table 2. We investigated the linkage disequilibrium (LD) of the *VEGFA* polymorphisms in our study population. As the high LD of the four neighboring loci, rs699947, rs833061, rs2010963 and rs25648, was an evidence for shared segregation, we performed a haplotype analysis (data not shown). To perform an in-depth analysis, haplotypes were estimated from the genotypes defined by loci rs699947, rs833061 and rs2010963. The frequencies of the three main groups of haplotypes in the CLL cohort were ACG (0.39), CTC (0.38) and CTG (0.21), respectively.

**Table 2:** Minor allele frequencies (MAF) of the *VEGF* polymorphisms studied in CLL patients.

Locus	Alleles	Frequencies
rs699947	C:A	0.45
rs833061	T:C	0.46
rs2010963	G:C	0.35
rs25648	C:T	0.15
rs3025039	C:T	0.13

All haplotypic and allelic frequencies were highly similar to those presented by a cohort of healthy blood donors from the same area (n = 183, data not shown). Moreover, because the majority of the participants of this study were of Spanish Caucasian origin, all frequencies fall in the same range of series reported in previous studies of other populations of white ethnicity [20], [28], [29].

### *VEGFA* genotypes and CLL survival

**Figure 1:**
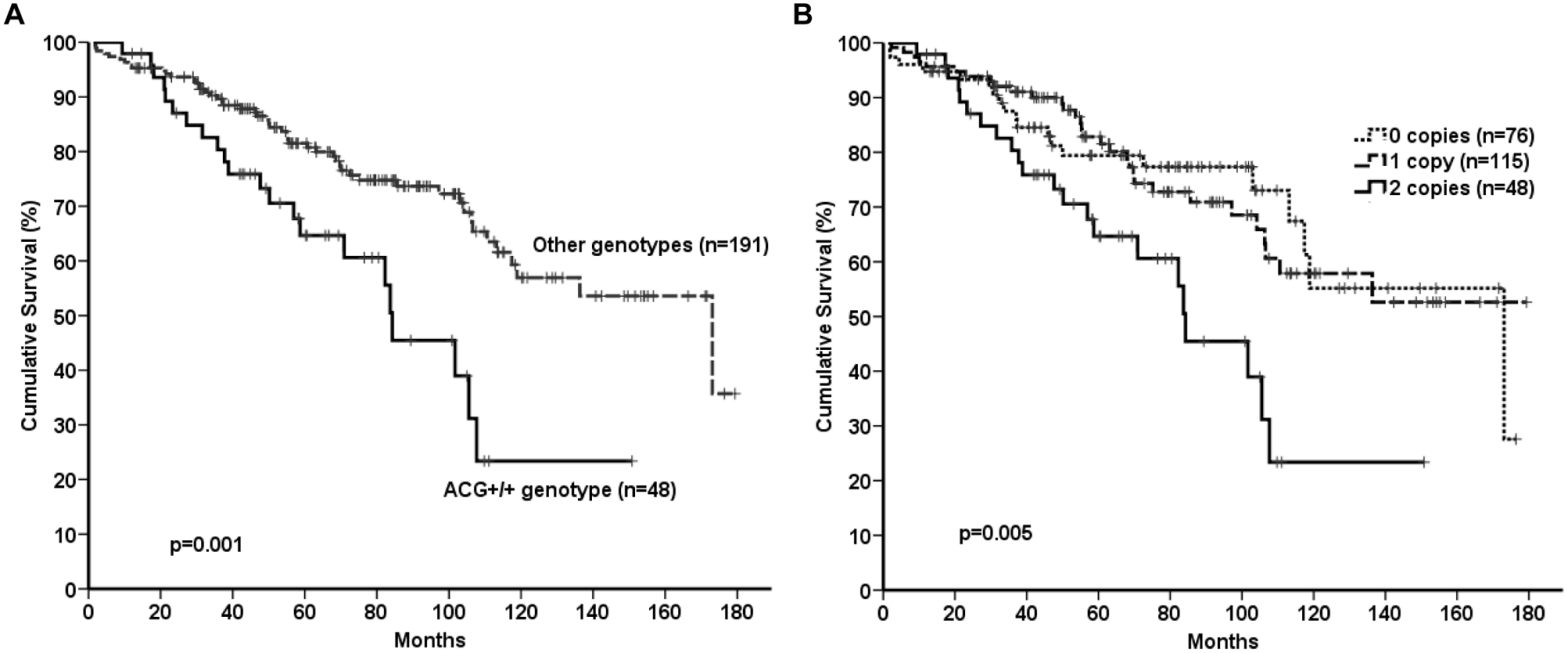
Association of *VEGFA* genotypes and OS of 239 CLL patients. Kaplan-Maier curves according to: (A) a recessive comparison of rs699947/rs833061/rs2010963 ACG^+/+^ genotype and, (B) number of copies of ACG haplotype.

We investigated the prognostic value of *VEGFA* variants in relation to patients’ overall survival (OS). For this, we monitored the entire cohort of 239 patients diagnosed from January 1997 to December 2010, with a median follow-up of 63.2 months (1.4–179.4) and a five-year survival of 77.4%. In this cohort, we studied the prognostic value of the *VEGFA* rs699947/rs833061/rs2010963 and rs3025039 and rs25648 polymorphisms (Fig. 1).

Comparison of their genotypes in a recessive genetic model showed that patients with the homozygous rs699947/rs833061/rs2010963 ACG genotype (ACG^+/+^) presented an increased mortality rate compared with patients with other genotypes (Fig. 1A). The median OS of patients with the ACG^+/+^ genotype was 84.3 months compared with 173.1 months (p = 0.001) for patients with other genotypes (ACG^×/−^ = 1 copy ACG [ACG^+/−^] + no copies ACG [ACG^−/−^]). The adverse effect observed for the ACG haplotype appears to be recessive because patients with 1 copy of the risk haplotype (ACG^+/−^ genotypes) and in the absence of the risk genotype (ACG^−/−^ genotypes) behaved similarly, and no significant difference in median OS was observed between both groups (not reached vs 173.1 months, p = 0.92, Fig. 1B).

In addition to *VEGFA* genetics, other previously reported variables were also significant epidemiological, clinical and molecular risk factors in OS prediction in our cohort: clinical stage (Binet), *IgV_H_* mutational status, *ATM* deletions, *TP53* disruptions, cytogenetic abnormalities, CD38 status and age at diagnosis (data not shown).

In the multivariate Cox regression model for OS, the conventional variables that showed an independent prognostic value were *IgV_H_* mutational status, *ATM* deletions, *TP53* deletions and age of diagnosis. Additionally, the *VEGFA* ACG^+/+^ genotype was identified as a novel prognostic parameter in OS, independent of other known factors (adjusted HR = 2.1, p = 0.005, Table 3).

**Table 3:** Multivariate analysis of the associations between *VEGFA*rs699947/rs833061/rs2010963 genotypes and OS of CLL patients.

Variable	Comparison	HR (95% CI)	p-value
Age at diagnosis^a^	>65 vs <65	2.7 (1.7–4.4)	7×10^−5^
Binet stage	B+C vs A	2.0 (1.2–3.4)	0.01
*TP53* deletion	Yes vs No	3.8 (2.3–6.5)	5×10^−7^
del11q	Yes vs No	1.9 (1.0–3.5)	0.051
*IgVH* genes	UM vs M	2.3 (1.3–4.3)	0.01
***VEGF*** **genotypes** b	**ACG^+/+^ vs others**	**2.1 (1.2–3.5)**	**0.005**

a Patients aged 65 year have been classified >65.

b Haplotype rs699947/rs833061/rs2010963.

HR: hazard ratio, Pos: positive, Neg: negative.

### Influence of VEGF genetic polymorphisms on clinical and molecular low-risk CLL subgroups

To identify protective or risk interactions, a review of the distribution of *VEGFA* variability in different subgroups of CLL was undertaken and revealed an asymmetric frequency of the ACG^+/+^ genotypes in patients with different mutational status of the *IgV_H_* genes (n = 222). The frequency of haplotype ACG defined by loci rs699947/rs833061/rs2010963 was 0.49 in the group of patients with poor prognosis (unmutated *IgV_H_* genes; UM-CLL) and 0.38 in patients with favourable prognosis (mutated *IgV_H_* genes; M-CLL). No asymmetries in the distribution of the haplotype ACG was found between groups defined by other epidemiological, clinical or molecular parameters.

Comparison in a recessive genetic model of the *VEGFA* rs699947/rs833061/rs2010963 genotypes (ACG+/+ vs ACG×/−) showed that homozygosity for the ACG haplotype was associated with forms of the disease with a worse prognosis. The ACG^+/+^ genotype was 2.5 times more common among individuals with unfavourable prognosis (UM-CLL) relative to other genotypes (OR = 2.5, two-sided p = 0.012). These recessive genetic associations do not denote an allele-dose effect as frequencies of heterozygous genotypes are not increased in the UM-CLL group (see Table 4). No statistical associations were found between the ACG^+/+^ genotype and other clinical or molecular variables analysed. Allele or genotype frequencies of polymorphisms rs3025039 and rs25648 also failed to associate with any of the analysed groups (data not shown).

**Table 4:** Distribution of *VEGF* genotypes in B-CLL groups with different mutational status of the*IgV_H_* genes.

Variant	Genotypes	M % (n) (N = 98)	UM %(n) (N = 124)	OR (95% IC)^b^	p-value
haplotype ACG^a^	X/X	35.7 (35)	29.0 (36)	1	
	ACG/X	52.0 (51)	45.2 (56)	1.1 (0.6–2.0)	0.83
	ACG/ACG	12.2 (12)	25.8 (32)	**2.5 (1.2–5.2)**	**0.012**
rs25648	C/C	72.4 (71)	70.2 (87)	1	
	C/T	26.5 (26)	29.0 (36)	0.9 (0.5–1.6)	0.69
	T/T	1.0 (1)	0.8 (1)	0.9 (0.5–1.6)	0.70
rs3025039	C/C	68.4 (67)	78.2 (97)	1	
	C/T	31.6 (31)	20.0 (26)	1.7 (0.9–3.2)	0.08
	T/T	0	0.8 (1)	1.7 (0.9–3)	0.1
					

M: patients with mutated *IgV_H_* genes, UM: patients with unmutated*IgV_H_* genes.

OR: odds ratio; CI, confidence interval.

a Haplotype rs699947/rs833061/rs2010963. X genotype corresponds to n haplotypes other than ACG (CTC or CTG).

b The upper homozygous genotype of each variant is designated the reference with an arbitrary OR value of 1 upon which the OR of the other genotypes are based.

Significant risk factors are shown in bold.

**Figure 2:**
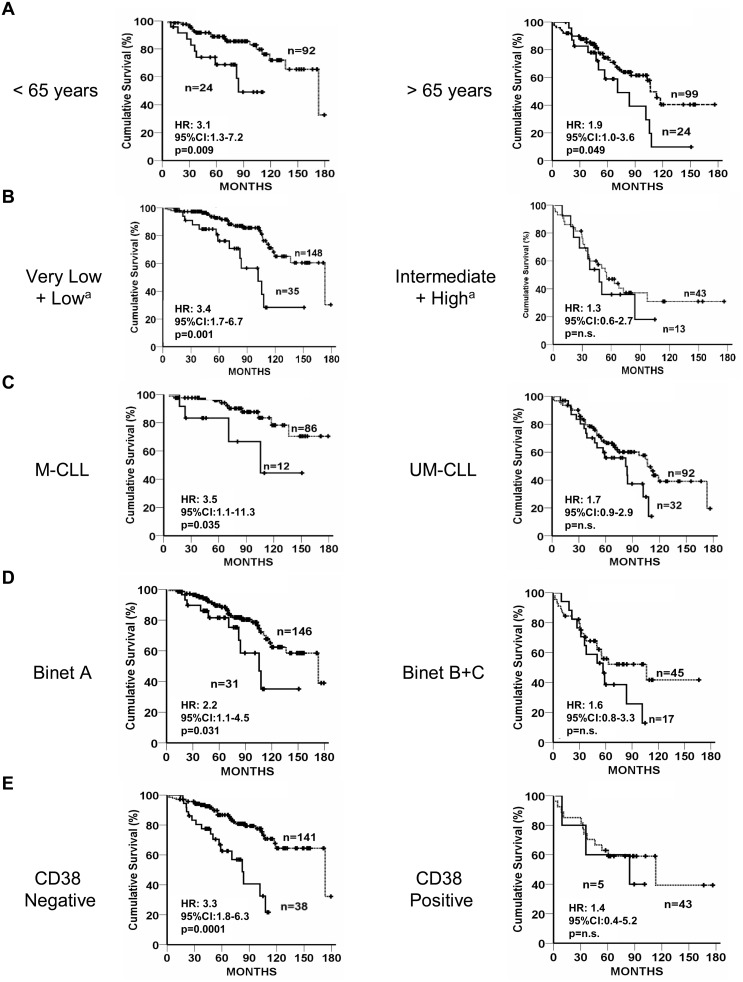
Association of *VEGFA* ACG+/+ genotype and OS of CLL patients with good prognostic features. Kaplan-Maier curves according to *VEGFA* ACG^+/+^ genotype (solid line) and other genotypes (dotted line) in subgroups of patients divided by (A) age at diagnosis, (B) *IgVH* mutational status, (C) Binet stage, (D) CD38 status and, (E) genetic abnormalities. ^a^Very low (del13q), low (normal karyotype), intermediate (Δ*ATM*) and high-risk (Δ*TP53*).

As the aforementioned ACG^+/+^ genotype was asymmetrically distributed in UM-CLL and M-CLL patients, we analysed the OS independently in the two groups. We did not observe any influence of the ACG^+/+^ genotype on the OS of the UM-CLL group, but conversely, in the group of patients with a favourable outcome (M-CLL), this genotype significantly correlated with a shorter survival (median OS: ACG^+/+^ = 105.5 months vs ACG^×/−^ = not reached; p = 0.025; Fig. 2). To further investigate a possible biological influence of this *VEGFA* genotype on the outcome of low-risk CLL, we performed the analysis within groups of patients defined by other markers of indolent course. We categorized our group of CLL patients by age at diagnosis, cytogenetic abnormalities, CD38 status or Binet stage (Fig. 2). Similarly to the subgroup with favourable*IgVH*mutational status, in patients with very low or low-risk genetics (deletion 13q, trisomy 12, normal karyotype) a decreased median OS from 173.1 to 101.7 months (p = 1.3×10^−5^) is associated with the presence of the ACG^+/+^ genotype. Again, the ACG^+/+^ genotype correlated with a reduction in the median OS from 173.1 to 105.5 months (p = 0.031) from 173.1 months to 82.3 months (p = 3.8×10^−5^) and from 173.1 to 84.3 (p = 0.006) in patients with an initial clinical stage (Binet A), negative expression of CD38 and age at diagnosis <65 years, respectively. In contrast, *VEGFA* genetics in the defined groups of poor outcome does not have any appreciable influence on the clinical course with the exception of the group of patients older than 65 years, in which the OS tended to be worse for those patients with the homozygous ACG^+/+^ genotype.

## Discussion

It has been extensively reported that angiogenesis mediated by the irregular expression of proangiogenic factors such VEGF by leukemic cells plays an important role in the pathogenesis of CLL. In this study, we analysed the contribution of *VEGFA* genetics in the clinical outcome of the leukaemia.

An analysis of the impact on disease progression of *VEGFA* SNPs in our population-based CLL cohort demonstrated that thers699947/rs833061/rs2010963 ACG genotype in homozygosity (ACG^+/+^) is a strong independent predictor of OS. The reduced survival observed in these patients could be related to increased angiogenesis as a result of an enhanced gene expression described for *VEGFA* variants rs699947, rs833061 and rs2010963. This would constitute a potential biological rationalization to the genetic association described in this work. Nonetheless, the justification to our findings could be due to other VEGF-mediated mechanisms of the pathogenesis of CLL. High-expressor *VEGFA* genotypes could increase the resistance to apoptosis, the cell motility or influence the microenvironment to generate more aggressive tumoral cells and consequently contribute to the progression of the leukaemia.

A further analysis of *VEGFA* genetics in CLL subgroups defined by different clinical and molecular parameters assessed an association between the *VEGFA* ACG^+/+^ genotype with a molecular marker of aggressive leukaemia: the unmutated status of the immunoglobulin variable gene segments (*IgV_H_*). *IgV_H_* mutation status parameter discriminates between leukaemia originating from B cells with an immunoglobulin gene submitted (mutated *IgV_H_*) or not (unmutated *IgV_H_*) to somatic hypermutation in the heavy chain variable region genes during ontogeny. Patients with a high number of mutations tend to have a better prognosis (M-CLL) than those with a low number (UM-CLL). Although the ACG^+/+^ genotype is less frequent among M-CLL patients, it correlated with a shorter overall survival within this category. A similar adverse effect of the ACG^+/+^ genotype in disease progression was found when we stratified patients with good prognostic features such as genetic abnormalities of favourable prognosis, Binet stage A, negative expression of CD38 or early age at diagnosis. These results suggest a potential clinical utility of *VEGFA* genetics as predictors of reduced survival in patients with indolent leukaemia and would allow to further refine the classification of this group of CLL patients. Although our study did not find strong differences in survival associated to the ACG^+/+^ genotype in patients with poor prognostic features, this observation might be perhaps more apparent than real. The large effect of other molecular factors in subgroups of poor outcome may be masking the differences in survival associated with *VEGFA* genotypes. Although we observed a large effect of *VEGFA* genotypes in the subgroup analyses, we were not able to analyse an independent cohort of CLL patients to confirm these differences in survival. In this manner, further studies in other CLL cohorts should be performed to validate these results and would be of relevant interest the analysis of *VEGFA* genetics and CLL in other ethnic groups. To date, exist few reports studying *VEGFA* polymorphisms as prognostic markers of CLL. A recent publication of a case-control study in a Polish CLL cohort [30], described an increased frequency of the rs3025039_allele T in patients with advanced stage (Rai III/IV) compared to healthy individuals.

A previous work by Pepper *et al* [31], found an anti-apoptotic over-expression of VEGF in CLL cells with high levels of CD38, which was associated to the reduced prognosis of the CD38-positive patients. However, we found a similar distribution of the *VEGFA* polymorphisms in patients with different CD38 expression levels indicating that the elevated VEGF expression of this subgroup is not a result of their *VEGFA* genetic background.

The *VEGFA* gene 5′-flanking region is highly variable and contains strongly linked polymorphisms. This increased diversity complicates the characterization of *VEGFA* haplotypes in the promoter region, but some of these linkages are consistently preserved and associated with disease and high *VEGFA* expression [18], [23]. Alleles A of rs699947 and C of rs833061 are associated with an upstream 18-nucleotide insertion that could modify the binding of factors in the *VEGFA* promoter. It has therefore been hypothesized that it could be responsible for the enhanced expression of this gene [20]. It would be interesting to perform further studies analysing the *VEGFA* levels in serum, the vascularity in the bone marrow and/or the resistance to apoptosis of CLL cells of patients with the VEGFA ACG+/+ genotype in order to explore the implication of this genotype in the physiopathology of the leukemia. A previous report [1], described an increased vascularity in the UM-CLL subset establishing subsequently a relation of a progressive phenotype of the leukaemia with an enhanced or aggressive angiogenic environment. This observation could be the result of the association of the UM-CLL subgroup with the high-expressor *VEGFA* ACG^+/+^ genotype that we have reported in this work.

The implication of the presence of increased vascularity in the bone marrow has been described in the outcome of various haematological diseases [32], [33]. This led to the study of the clinical significance of *VEGFA* genetics in other hematologic malignancies. In acute myeloid leukaemia, the rs699947/rs833061/rs2010963 CTG haplotype and rs3025029 C/C genotype influence the leukaemia-free survival (LFS), event-free survival (EFS) and overall survival (OS) of patients [34]. In chronic myeloid leukaemia, the rs699947 A/A and rs833061 C/C genotypes correlate with progression to advanced disease [35]. *VEGFA* polymorphisms also associate with clinical parameters in non-Hodgkin’s lymphoma (NHL). In these patients, rs699947 CA and CA + AA genotypes and rs3025039 TT genotypes, as well as, rs699947 A and rs3025039 T alleles are associated with decreased risk for invasion [36]. Furthermore, rs3025039 T allele was found more frequently in NHLs with worse prognosis (patients with International Prognostic Index (IPI)-3 and/or 4) [37].

Elevated serum levels of its receptor, VEGF-R2 were also associated with lymphocytosis, severe anaemia and shortened survival in CLL [14]. These results also support the role of the VEGF system in this leukaemia and suggest a potential benefit in evaluating polymorphisms in *VEGF-R2* that might have an impact on CLL outcome.

It has been shown that disruption of proangiogenic factors can inhibit tumour vessel density and growth, and the combination of anti-angiogenic agents with other drugs has certain synergetic effects in improving the OR rate in solid tumours. Although, results of the first clinical trials evaluating anti-VEGF therapy for patients with CLL indicate a lack of efficacy in patients with relapsed/refractory CLL [38], combining VEGF blocking with conventional treatments may be a potential therapeutic approach for patients with CLL. In addition to the hypothetical use of *VEGFA* SNPs to predict the clinical outcome for CLL, these polymorphisms could be used as markers of clinical efficacy for chemoimmunotherapy with novel agents targeting VEGF or its receptor (VEGFR) to prevent leukaemia progression [39].

In conclusion, *VEGFA* genetic diversity appears to influence the physiopathology of CLL. The genotyping of polymorphisms in this gene can be performed easily using a rapid PCR method and, together with other parameters, these genetic markers might be utilized both in clinical practice and in further studies of CLL to upgrade patients’ prognosis and select target therapies.
